# Allergy influences the inflammatory status of the brain and enhances tau-phosphorylation

**DOI:** 10.1111/j.1582-4934.2012.01556.x

**Published:** 2012-09-26

**Authors:** Heela Sarlus, Caroline Olgart Höglund, Bianka Karshikoff, Xiuzhe Wang, Mats Lekander, Marianne Schultzberg, Mircea Oprica

**Affiliations:** a Division of Neurodegeneration Department of Neurobiology Care Sciences and Society, Karolinska InstitutetStockholm, Sweden; b Respiratory Medicine Unit Lung Research Laboratory, L4:01 Department of Medicine Solna, Karolinska Institutet and Karolinska University Hospital SolnaStockholm, Sweden; c Section for Neuroimmunology Department of Physiology and Pharmacology, Karolinska InstitutetStockholm, Sweden; d Stress Research Institute, Stockholm UniversityStockholm, Sweden; e Osher Center for Integrative Medicine and Center for Allergy Research, Karolinska InstitutetStockholm, Sweden; f Department of Neurology, Karolinska University HospitalStockholm, Sweden

**Keywords:** immunoglobulin, neuroinflammation, tau-phosphorylation, Alzheimer's disease

## Abstract

Despite the existing knowledge regarding the neuropathology of Alzheimer's disease (AD), the cause of sporadic forms of the disease is unknown. It has been suggested that systemic inflammation may have a role, but the exact mechanisms through which inflammatory processes influence the pathogenesis and progress of AD are not obvious. Allergy is a chronic inflammatory disease affecting more than 20% of the Western population, but the effects of allergic conditions on brain functions are largely unknown. The aim of this study was to investigate whether or not chronic peripheral inflammation associated with allergy affects the expression of AD-related proteins and inflammatory markers in the brain. On the basis of previously described models for allergy in mice we developed a model of chronic airway allergy in mouse, with ovalbumin as allergen. The validity of the chronic allergy model was confirmed by a consistent and reproducible eosinophilia in the bronchoalveolar lavage (BAL) fluid of allergic animals. Allergic mice were shown to have increased brain levels of both immunoglobulin (Ig) G and IgE with a widespread distribution. Allergy was also found to increase phosphorylation of tau protein in the brain. The present data support the notion that allergy-dependent chronic peripheral inflammation modifies the brain inflammatory status, and influences phosphorylation of an AD-related protein, indicating that allergy may be yet another factor to be considered for the development and/or progression of neurodegenerative diseases such as AD.

## Introduction

Systemic inflammation has been shown to worsen the progress of Alzheimer's disease (AD) [1]. Elevated plasma levels of inflammatory proteins have been detected before clinical onset in patients with AD and mild cognitive impairment (MCI) [2, 3], suggesting that chronic inflammation may be involved in the initiation and progress of the disease. AD is a neurodegenerative disorder characterized by progressive dementia with devastating effects for the patients and their families. The cause of the sporadic form of the disease accounting for more than 95% of the cases is unknown while available treatments are purely symptomatic. Therefore, it would be of great value to find treatment strategies aiming at the etiopathogenesis of AD. The theory of immunopathogenesis of AD has obvious therapeutic implications. Firstly, retrospective studies have shown decreased AD prevalence and progression rate in elderly patients with history of long-term anti-inflammatory therapy [4]. Secondly, immunomodulatory strategies in animal models have been shown to result in promising effects on AD-related behavioural and pathological features [5–8]. Thus, the notion that inflammation represented in the periphery gives rise to central manifestations related to the pathophysiology in AD, could represent an opportunity to modulate a factor involved in disease pathogenesis.

Despite the existing knowledge regarding the neuropathology of the disease, the cause of AD is not known. The neuropathological hallmarks of AD are the extracellular amyloid deposits, comprised mainly of β-amyloid (Aβ) peptide, and the intracellular neurofibrillary tangles (NFTs), consisting of hyperphosphorylated tau protein [9]. Reduced synaptic density and neuronal loss are also part of the neuropathology in the AD brain. The extent of NFTs primarily found in brain regions that are critical for memory [10] seems to better correlate with the severity of dementia in humans than the amyloid plaques [10–12]. It has been hypothesized that NFTs are responsible for impairing synaptic function, leading to cognitive malfunction [13]. The level of tau-phosphorylation is regulated dynamically by several kinases and phosphatases. Indeed, analysis of AD brain tissue showed that protein kinases such as glycogen synthase kinase 3β (GSK3β), P25/Cyclin-dependent kinase 5 (Cdk5), as well as mitogen-activated protein kinases (MAPKs), such as extracellular signal-regulated MAP kinase (ERK) ½ pathway, the c-Jun-N-terminal kinase (JNK) pathway, and the p38 pathway, are increased in expression and/or activity [14]. Decreased activity is observed for protein phosphatases (PPs) such as PP1, PP2A and PP5 [15]. Therefore, an imbalance between the activities of kinases and phosphatases may cause tau-hyperphosphorylation, which may be related to clinical symptoms encountered in AD.

A large body of evidence suggests that inflammatory processes in the brain have an important, but yet not obvious role in the initiation and/or progression of AD [16, 17]. The inflammatory component in AD consists of microglial activation followed by astroglial proliferation, with the production of several inflammatory proteins, such as pro-inflammatory cytokines, chemokines, complement factors and acute phase reactants. An increased expression of pro-inflammatory cytokines such as interleukin (IL)-1 has been found in the human AD brain [18], and transgenic mice with AD-pathology have increased expression of IL-1 and IL-6 in the brain [19, 20]. Decreased levels of the endogenous IL-1 receptor antagonist (IL-1ra) in cerebrospinal fluid (CSF) from AD patients may indicate an imbalance between IL-1 and IL-1ra in the AD brain [21], whereas the increased levels of the soluble IL-1 receptor type II (sIL-1RII) [22] may suggest an attempt to balance the inflammation.

Allergy is a highly prevalent peripherally manifested chronic inflammatory condition affecting around 20% of the adult population, with onset often already in childhood or adolescence. A potential connection between allergic disorders and AD has been suggested, a recent epidemiological study showed for the first time that a history of atopy is associated with an increased risk for developing AD [23].

The effects of allergic conditions on brain functions are largely unknown. A few studies have been performed on mice challenged with ovalbumin (OVA), an allergen widely used in murine models of allergy. OVA challenge in mice was shown to increase *c-fos* expression in the brain [24], and enhance anxiety-related behaviours [25], suggesting that allergy influences brain activity. There are indications that allergy is associated with an induction of pro-inflammatory cytokines in the brain. Increased levels of the cytokines IL-1α and tumour necrosis factor (TNF)-α have been demonstrated in the brain of OVA-sensitized mice, after exposure to polluted air particles [26]. A recent study demonstrated that experimental models of allergic rhinitis are associated with a Th2 pattern of cytokine mRNA expression in the brain [27]. Thus, a potential link between allergy, brain inflammation and AD seems to be worth exploring.

The purpose of the present work was to study the effects of chronic allergy on markers for inflammation and AD neuropathology, including immunoglobulins, inflammatory cytokines, tau-phosphorylation and *β*-amyloid precursor protein (APP) in the brain in a model of chronic allergy in mice, to better elucidate the mechanisms behind a potential connection between two highly prevalent diseases, AD and allergy.

## Materials and methods

### Animals

Male 12–14 weeks old Balb/c (20–24 g, *n* = 60) and C57B6 (20–22 g, *n* = 45) mice were purchased from B&K Sollentuna AB, Sweden. The animals were housed 3–5 per cage under controlled conditions of light-dark cycle (12:12 hrs, lights on at 06:00 hrs), temperature (21 ± 1°C), relative humidity (60–65%) and food and water *ad libitum*. Upon arrival, the animals were habituated to the environment for two weeks before the start of experiments, and handled daily for minimizing the stress level after the start of the chronic allergy protocol. The study was approved by Stockholm South local committee on ethics of animal experiments (S200/07).

### Allergen provocation protocol, tissue collection and bronchoalveolar lavage

**Fig 1:**
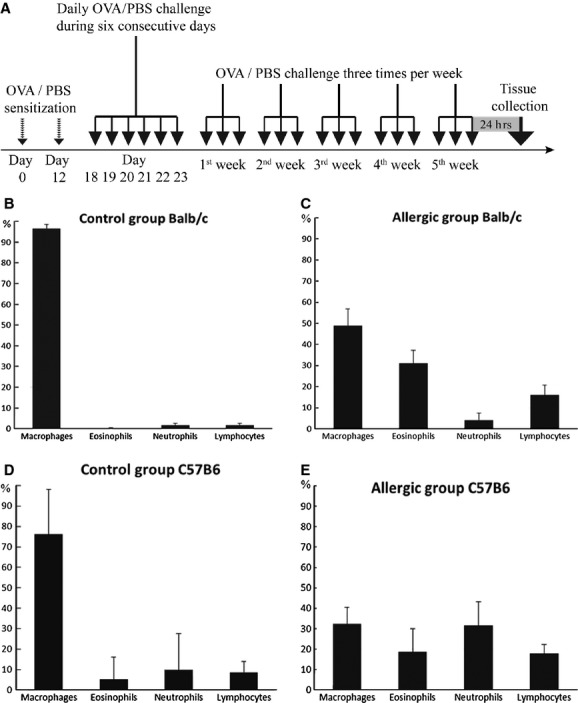
(A–E) Balb/c and C57B6 mice were sensitized to ovalbumin (OVA) given as a single intraperitoneal (i.p.) injection on day 0 and 12 (A). The animals were then challenged daily from day 18 to day 23, and then three times per week during an additional 5-week period (A). Eosinophilic granulocytes were increased in bronchoalveolar lavage (BAL) fluid, with regard to the total number of cells in allergic Balb/c animals (C) as compared to control mice (B), and in C57B6 allergic mice (E) as compared to control mice (D). Allergic C57B6 mice (E) had higher proportion of neutrophilic granulocytes in the BAL fluid than Balb/c mice (C).

Both AD and allergy are chronic disorders, and we have therefore validated a modified version of a previously described chronic model of airway-induced allergy in Balb/c (*n* = 30, 15 allergics and 15 controls) and C57B6 (*n* = 15, 7 allergics and 8 controls) mice, using a chronic OVA challenge protocol [28] (Fig. 1A). Balb/c mice are prone to develop allergic reactions with high numbers of eosinophils and high IgE expression, and it is the most widely used strain in experimental allergy models [29]. The C57B6 mouse strain was chosen because it is frequently used as background strain for transgenic mouse models for AD. The biochemical and immunohistochemical experiments described in the present study were performed on other groups of Balb/c (*n* = 30) and C57B6 (*n* = 30) mice subjected to the chronic airway-induced model of allergy described below. For each of the mouse strains, 20 mice (10 allergics and 10 controls) were allocated for biochemical studies, and 10 mice (five allergics and five controls) were allocated for immunohistochemical studies.

Mice were sensitized with 10 μg OVA grade III (Sigma-Aldrich, St. Louis, MO, USA) diluted in a suspension of 4 mg/ml Al(OH)_3_ in phosphate-buffered saline (PBS). A single intraperitoneal (i.p.) injection of 200 μl suspension was given on day 0 and 12. The animals were then challenged daily from day 18 to day 23, and then three times per week during an additional 5-week period, by intranasal instillation of 100 μg OVA diluted in 50 μl PBS (Fig. 1A). Intranasal instillation was performed under light anaesthesia with a controlled flow of 4% isoflurane and 96% O_2_, using a Univentor 400 anaesthesia unit. Control animals were given PBS instead of OVA, but otherwise underwent the same treatment and were handled identically.

The animals were killed 24 hrs after the last antigen challenge, and tissues collected for biochemical and immunohistochemical studies. The hippocampus and parietal cortex were quickly dissected out, frozen on dry ice and stored at −70°C until processed for biochemical studies including immunoblotting and multiplex cytokine assay. Mice assigned for immunohistochemistry were perfused as described below.

After brain tissue collection, the lungs were dissected out and the trachea was cannulated with a catheter. The lungs were carefully flushed twice with a total of 550 μl ice-cold PBS (pH 7.4), during which the lobes were manually massaged so that the lungs were evenly filled. The recovery rate was 300 μl on average. The BAL fluid was centrifuged at 400 × *g* for 10 min. at 4°C, and the pelleted cells were harvested and resuspended in cold PBS, and then recentrifuged at 900 × *g*. The total cell count was assessed using a Bürker chamber. For differential cell counting, the cells were spinned onto glass slides, air-dried, fixed in ethanol, and stained with the May-Grünwald/Giemsa method. The number of eosinophils, macrophages, neutrophils and lymphocytes were counted on the basis of morphology.

### Biochemical studies

The brain tissues were homogenized by sonication in homogenization buffer consisting of 20 mM Tris-HCl, pH 6.8, containing 137 mM NaCl and 2 mM EDTA, and supplemented with a protease inhibitor cocktail (Sigma-Aldrich), phosphatase inhibitors (HaltTM cocktail; Pierce, Bradford, IL, USA), and 2 nM okadaic acid (Sigma-Aldrich). The brain tissue homogenates from Balb/c mice were first centrifuged at 4°C for 1 hr at 100,000 × *g* in the absence of detergents. The supernatant representing the cytosolic fraction was collected and rapidly frozen. The pellet was resuspended in homogenization buffer in the presence of detergents, 2% NP-40 and 2% Triton X (Sigma-Aldrich), followed by centrifugation at 4°C for 1 hr at 100,000 × *g*. The supernatant representing the membrane fraction was collected and rapidly frozen. In the case of brain tissues from the C57B6 mice, the homogenization was performed in one step in the same homogenization buffer, but with addition of detergents. The homogenates were centrifuged at 11,000 × *g* at 4°C for 20 min., and the supernatants collected and rapidly frozen. The protein content of the supernatants was determined using a BCA kit (Sigma-Aldrich).

The different homogenization protocols were used for optimization of the measurements of cytokines and for isolation, in the case of Balb/c mice, of material needed for analysis of several membrane-bound proteins not related to the present study. The results for cytokine measurements are reported for the C57B6 mice, as high speed homogenization used in the case of Balb/c mice may cause cytokine damage (M. Oprica, M. Schultzberg, unpublished observations).

### Immunoblotting

Analysis of the expression of immunoglobulins (Igs), APP, tau-protein, and tau-phosphatases, was performed by immunoblotting of samples from allergic Balb/c and C57B6 (*n* = 9 for each strain) and control mice (*n* = 9 for each strain). One allergic and one control mouse, out of ten for each of the treatment groups, were randomly excluded from immunoblotting studies due to limited space on the running gels. Samples containing equal amounts of proteins (20–40 μg) were added to sodium dodecyl sulphate (SDS) in sample loading buffer 2X (50 mM Tris, pH 6.8, containing 10% glycerol, 0.1% bromophenol blue, 2% SDS, and 5% β-mercaptoethanol; Sigma-Aldrich), electrophoresed in 10% SDS-polyacrylamide gel, and transferred to a 45 μm nitrocellulose membrane (Bio-Rad Laboratories, Inc., Hercules, CA, USA). After blocking with 5% non-fat milk in TBS-T (20 mM Tris-HCl, pH 7.6, 137 mM NaCl and 0.1% Tween 20) for 30 min. at 20°C, the membrane was incubated overnight at 4°C with primary antibodies in blocking solution (see Table 1). The blots were washed with TBS-T for 30 min., and incubated with secondary antibodies (GE Healthcare, Buckhinghamshire, UK; Jackson Laboratories, Bar Harbor, ME, USA) for 1 hr at 20°C. After rinsing in TBS-T for 30 min., the blots were incubated for 5 min. with ECL (GE Healthcare), and the chemiluminiscence was detected using a CCD camera (Las-3000; Fujifilm, Tokyo, Japan). Quantification of the intensity of the bands obtained was performed with the Multi Gauge (V 3.0) software (Fujifilm). All the proteins analysed were normalized to α-tubulin, used as house-keeping protein except for P-Tau which was normalized to total tau.

**Table 1:** Antibodies used for biochemical and immunohistochemical studies

Primary antibodies	Dilution	Source
IgG	1:1000	GE Healthcare
IgE	1:2000	Abcam
AT180	1:1000	Innogenetics
AT8	1:1000	Innogenetics
T-tau	1:3000	Abcam
Cdk5	1:5000	Santa Cruz
p35/p25	1:2000	Santa Cruz
p-ERK 1/2	1:2000	Cell Signalling
ERK-pan	1:5000	BD transgenics
p-GSK3β S9	1:1250	Cell signalling
p-GSK3β Y216	1:2000	Invitrogen
GFAP	1:1000	Invitrogen
APP	1:3000	Zymed laboratories
p-JNK	1:1000	Cell signalling
JNK-total	1:1000	Cell signalling
p-38	1:1000	Cell signalling
α-tubulin	1:15000	Sigma

### Multiplex cytokine assay

The levels of cytokines with relevance for pro-inflammatory, Th1 and Th2 types of immune responses [interferon (IFN)-γ, IL-1β, IL-2, IL-4, IL-5, IL-8, IL-10, IL-12, TNF-α] were analysed by using the Mesoscale 9-Plex assay Th1/Th2 kit for mouse according to the protocol supplied by the manufacturer (MSD; Meso Scale Discovery, Gaithersburg, MD, USA) in allergic (*n* = 10) and control (*n* = 10) C57B6 mice. The lower detection limit of the kit is different for each cytokine, and as given by the manufacturer: 0.47 pg/ml for IFN-γ, 2.1 pg/ml for IL-1β, 3.0 pg/ml for IL-2, 0.87 pg/ml for IL-4, 0.70 pg/ml for IL-5, 2.9 pg/ml for IL-8, 11 pg/ml for IL-10, 5.3 pg/ml for IL-12, 1.0 pg/ml for TNF-α.

### Immunohistochemistry

Analysis of IgG and IgE in the brain Balb/c mice (allergics, *n* = 3 and controls *n* = 4) as well as in C57B6 mice (allergics *n* = 4 and controls *n* = 4) was performed by immunohistochemistry. The difference from five animals per group, *i.e*. allergics and controls initially allocated for immunohistochemical studies for both mouse strains, is due to animals lost during tissue processing. The animals were perfused intracardially with 20 ml 0.01 M PBS, pH 7.4, followed by 50 ml 4% paraformaldehyde (PF) in 0.1 M Sörensen phosphate buffer, pH 7.4, containing picric acid. The brain from both mouse strains and spleen from C57B6 mice were dissected out, post-fixed for 2 hrs at 4°C in the same fixative, and then soaked in 10% sucrose in 0.1 M Sörensen phosphate buffer, pH 7.4, until further processed. The tissues were snap-frozen and sectioned in the coronal plane (12 μm thickness) using a cryostat (HM 350; Microm, Heidelberg, Germany). The sections were stored at −20°C until further processing.

Prior to the staining procedure, the sections were air-dried for 5 min. at room temperature (RT) and rinsed in PBS, pH 7.4. For detection of mouse IgG, the sections were incubated for 30 min. with 5% normal horse serum (Jackson ImmunoResearch, Suffolk, UK) in 0.3% Triton X100/PBS, and then incubated for 90 min. at RT with biotinylated horse antimouse IgG antibodies (1:200; Vector Laboratories, Burlingame, CA, USA). Following rinsing in PBS, the sections were incubated with a streptavidin-HRP complex (ABC Vectastain kit; Vector Laboratories) for 30 min. at RT. The immunoreactivity was visualized by incubation with 1 mg/ml diaminobenzidine (DAB) in the presence of 3% H_2_O_2_ for 3 min. After rinsing, the sections were dehydrated in ethanol and mounted. For IgE staining, the sections were incubated for 30 min. with 5% normal rabbit serum (Jackson ImmunoResearch, Suffolk, UK) in 0.3% Triton X100/PBS. The sections were then incubated overnight at 4°C with the IgE antibodies (diluted 1:3000) used in immunoblotting (see Table 1), and then incubated with biotinylated rabbit anti-goat antibodies (Vector Laboratories) for 1.5 hrs at RT. The rest of the procedure was the same as for the IgG staining. The same protocol was employed for staining of microglia, using an antibody of rat antimouse F4/80 antibodies (diluted 1: 100; AbD Serotec, Mountain View, CA, USA). For astrocyte and plasma cell staining, the sections were incubated with 5% normal donkey serum (Jackson ImmunoResearch), followed by overnight incubation with rabbit GFAP antibodies (dilution 1:500; Dako, Glostrup, Denmark), respectively, rat CD138 antibodies (dilution 1:200; BD Biosciences, Franklin Lakes, NJ, USA) at 4°C. Subsequently, the sections were incubated for 1 hr at room temperature with secondary antibodies, FITC-conjugated donkey anti-rabbit IgG (dilution 1:400; Jackson), and Cy3-conjugated donkey anti-rat IgG (dilution 1:600; Jackson), respectively. Sections of the spleen were stained with CD138 antibodies by the same method.

The slides were analysed under a microscope (Nikon Eclipse E800, Nikon, Tokyo, Japan) at different magnifications and photographed.

### Statistics

The statistical analysis was performed using the Statistica Software v 9.1 (Statsoft Inc., Tulsa, OK, USA). Data on the levels of cytokines were analysed by Student's *t*-test. The estimation of cell counts in the BAL fluid and the immunoblot data were analysed by the Mann–Whitney test.

## Results

### Validation of the chronic airway-induced allergy model

To study the effects of peripheral inflammation associated with allergy on the brain we have developed a model of chronic airway-induced allergy (Fig. 1A), and validated the model in two mouse strains, Balb/c (Fig. 1B and C) and C57B6 mice (Fig. 1D and E). The efficiency and reproducibility of this chronic allergy model was first confirmed in Balb/c mice by a reproducible and consistent increase of eosinophilic granulocytes in relation to the total number of cells in allergic mice (*n* = 14), as compared to controls (*n* = 13) (Fig. 1B and C). The processing of BAL fluid failed for one allergic mouse and two controls. We have found significant increases both in the total number of cells (*P* < 0.001, *Z* = 4.003) and of eosinophils (*P* < 0.001, *Z* = 4.40) (see Fig. S1A, Supporting information) in the BAL fluid of allergic animals when compared with the BAL fluid from control animals. The C57B6 mice subjected to the allergy model (*n* = 7) also had higher numbers of eosinophilic granulocytes in the BAL fluid, as compared to control mice (*n* = 8) (Fig. 1D and E). In addition, allergic C57B6 mice had higher numbers of neutrophilic granulocytes in the BAL fluid as compared to controls (Fig. 1D and E), also shown in a previous study [30]. We have found significant increases both in total number of cells (*P* < 0.01, *Z* = 2.90) and in the number of eosinophils (*P* < 0.01, *Z* = 2.64) (see Fig. S1B, Supporting information) in the BAL fluid of allergic C57B6 mice when compared with control animals.

### Inflammatory markers

#### Immunoglobulins

**Fig 2:**
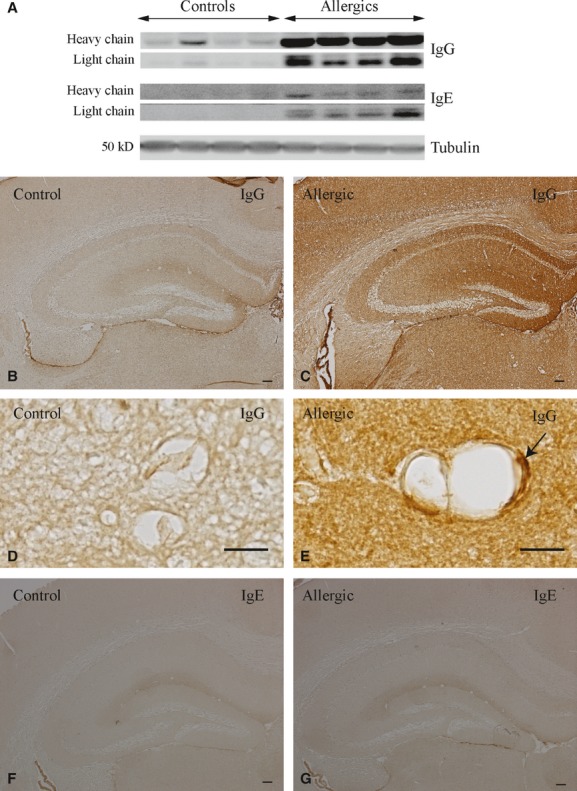
(A–G) The effects of chronic airway-induced allergy on the brain levels of IgG (A–E) and IgE (A, F, G) as shown by immunoblotting (A) and immunohistochemistry (B–G) in Balb/c mice. The results from immunoblotting show higher levels of IgG and IgE in the hippocampus (A) of the allergic animals as compared to control animals. The results from immunohistochemistry are in concordance with the immunoblotting results. Immunoreactive labelling for IgG is markedly enhanced in the brain of allergic animals (C and E), as compared to control animals (B and D). The IgG labelling is widespread in the brain parenchyma (C), and can also be seen in blood vessels upon allergy (arrow in E), and not in control animals (D). IgE immunoreactivity is also higher in the allergic animals (G) as compared to controls (F). The bars in B, C, F and G indicate 100 μm, and in D and E indicate 10 μm.

The levels of IgG and IgE were analysed by immunoblotting in the hippocampus and by immunohistochemistry in the brain of allergic and control Balb/c (Fig. 2A–G) and C57B6 mice (see Fig. S2A–E, Supporting information). Immunoblotting in the hippocampus of Balb/c (Fig. 2A) and C57B6 mice (Fig. S2A, supporting information) showed that allergic mice had higher levels both of IgG (*P* < 0.001, *Z* = −3.53 and, respectively, *P* < 0.001, *Z* = −3.63, data not shown), and of IgE (*P* < 0.001, *Z* = −3.53 and, respectively, *P* < 0.01, *Z* = −2.8, data not shown).

Immunohistochemical studies performed in Balb/c mice and C57B6 mice confirmed the results obtained by immunoblotting. Immunoreactive labelling for IgG was markedly enhanced in the brain of the allergic animals, as compared to control animals both in Balb/c (Fig. 2B–E) and C57B6 mice (see Fig. S2B–E, Supporting information). The increase in the IgG immunoreactivity was somewhat less marked in C57B6 mice (Fig. S2, Supporting information), than that seen in the Balb/c mice. IgG immunoreactivity was not only widespread in the brain parenchyma (Fig. 2C), but was also localized to blood vessels (Fig. 2E). IgE immunoreactivity was also increased in the allergic Balb/c mice, as compared to that in controls (Fig. 2F and G).

**Fig 3:**
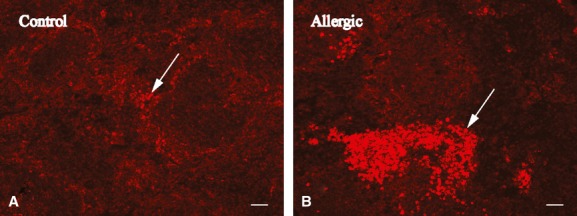
(A–B) The effects of chronic airway-induced allergy on the presence of plasma cells in the spleens of C57B6 mice. A higher number of CD138-positive plasma cells can be seen in the spleen of allergic animals (arrow in B), as compared with control mice (arrow in A). The bars indicate 50 μm.

To study the origin of IgG and IgE in the brain we analysed the occurrence of CD138 labelled cells. CD138 is a member of the syndecan family, which under normal conditions is predominantly expressed on mature Ig-secreting plasma cells [31]. No immunoreactivity could be seen for this marker, neither in the brain of allergic animals nor in control brains (data not shown). However, a higher number of CD138-positive cells were observed in the spleens of allergic C57B6 mice as compared to that in control mice (Fig. 3A and B).

#### Cytokines and cellular markers

Tissue samples of the parietal cortex and hippocampus of allergic and control C57B6 animals were analysed with a cytokine multiplex assay. There were no statistical differences in the levels of these cytokines between allergic and control animals, neither in the hippocampus (see Fig. S3A, Supporting information) nor in the parietal cortex (see Fig. S3B, Supporting information). The measured values found to be under the detection level of the kit were not considered in the analysis (see legend for Fig. S3A–B, Supporting information for details). IL-4 was not detectable in the parietal cortex.

To further examine the inflammatory status of the brain, markers for astrocytes (GFAP) and microglia (F4/80) were used. There was no difference in the extent of labelled cells in the brain between the allergic and control animals, neither for GFAP (see Fig. S4, Supporting information, showing an immunoblot for GFAP in the hippocampus), nor for F4/80 (data not shown).

#### AD markers

We further studied the effects of peripheral inflammation associated with chronic allergy on the AD-related proteins, tau and APP.

*Tau-phosphorylation:* It is well known that hyperphosphorylation of the tau-protein plays an important role in AD pathogenesis, and as mentioned, is more strongly correlated to the cognitive decline in AD patients than the amyloid-pathology is. To examine whether or not airway-induced peripheral inflammation affects tau-phosphorylation in the brain, the levels of phosphorylated-tau (P-tau) were analysed with the AT8 antibody that recognizes the phosphorylation sites at serine 199 and 202, and threonine 205, and the AT180 antibody that recognizes phosphorylated threonine 231 and serine 235.

**Fig 4:**
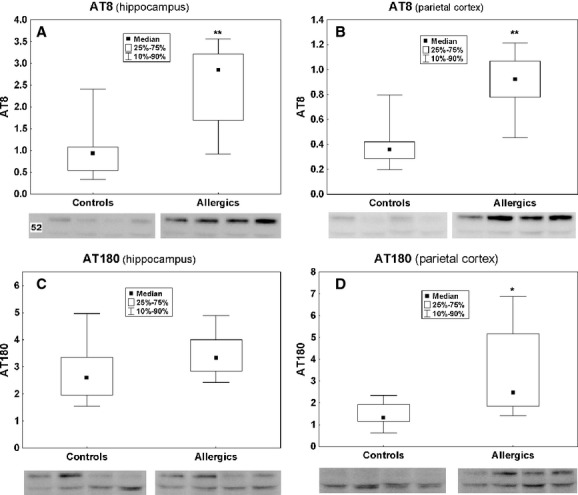
(A–D) The effects of chronic airway-induced allergy on tau-phosphorylation at the AT8 (A and B) and AT180 (C and D) phosphorylation sites in the hippocampus (A and C) and parietal cortex (B and D) of Balb/c mice. The phospho (P)-tau levels at the AT8 phosphorylation sites are higher in the allergic animals than those in the controls, both in the hippocampus (*P* < 0.01) (A) and in the parietal cortex (*P* < 0.01) (B). The levels of P-tau at the AT180 phosphorylation sites are significantly increased in the parietal cortex (*P* < 0.05) (D) of the allergic mice, but not in the hippocampus (C).

Significantly higher levels of P-tau were observed upon chronic allergy at both the AT8 and AT180 phosphorylation sites in Balb/c (Fig. 4A–D) and C57B6 (Fig. S5A–D, Supporting information) mice. The levels of P-tau at the AT8 phosphorylation sites were higher in the allergic Balb/c animals as compared to those in controls, both in the hippocampus (*P* = 0.01, *Z* = −2.38), and parietal cortex (*P* < 0.01, *Z* = −2.06) (Fig. 4A and B). The allergic C57B6 animals also had higher AT8 phosphorylation levels, both in the hippocampus (*P* < 0.05, *Z* = −2.00) (see Fig. S5A, Supporting information), and the parietal cortex (*P* < 0.05, *Z* = −2.81) (see Fig. S5B, Supporting information).

The levels of P-tau at the AT180 phosphorylation sites, were significantly increased in the parietal cortex (*Z* = −2.29, *P* < 0.05) of allergic Balb/c animals, but not in the hippocampus (Fig. 4C and D), although the trend was similar. In C57B6 mice, the levels of P-tau at the AT180 phosphorylation sites, were significantly increased both in the hippocampus (*Z* = −2.65, *P* < 0.01) (see Fig. S5C, Supporting information) and the parietal cortex (*Z* = −2.24, *P* < 0.05) (see Fig. S5D, Supporting information). There was no difference in the levels of total tau (T-tau) protein between allergic and control animals, neither in Balb/c nor in C57B6 animals (data not shown).

**Fig 5:**
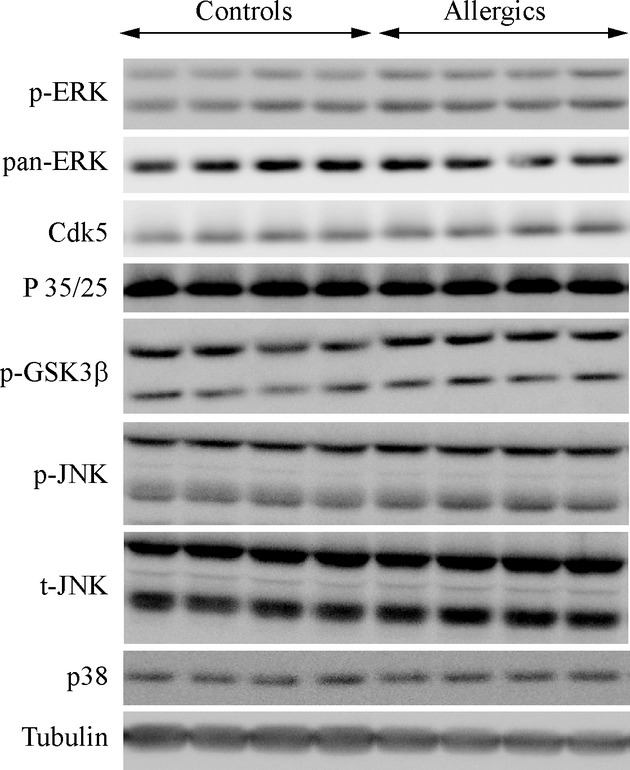
The effects of chronic airway-induced allergy on the expression of kinases involved in tau-phosphorylation in the hippocampus of Balb/c mice. There is no difference between the allergic and control animals for any of the examined kinases. The last blot shows the levels of α-tubulin used as housekeeping protein in the same animals.

*Tau-kinases:* To investigate the pathways involved in the observed increase in P-tau, the levels of certain kinases involved in tau-phosphorylation were analysed in samples of hippocampus and parietal cortex. There was no difference between allergic and control animals, neither in Balb/c (Fig. 5) nor in C57B6 (data not shown) mice, with regard to the hippocampal levels of p-ERK, pan-ERK, cdk5, p35/25, GSK3β, p-JNK, t-JNK and p38. The results were similar in the parietal cortex of the Balb/c mice (data not shown), as well as in the hippocampus and parietal cortex of C57B6 mice (data not shown).

**Fig 6:**
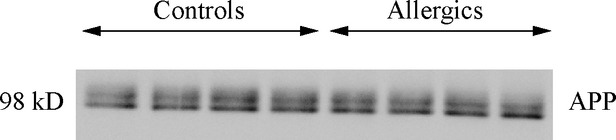
The effects of chronic airway-induced allergy on the expression of β-amyloid precursor protein (APP) in the hippocampus of Balb/c mice. No difference in the APP levels can be observed between the allergic and control animals.

*β-Amyloid precursor protein:* According to the amyloid hypothesis, increased Aβ levels in the brain derived from endoproteolysis of APP have a central role in AD pathogenesis. Analysis of the APP levels in the present allergy model, performed with an antibody directed against a 22 amino acids long region located at the COOH-terminus of APP, did not reveal any differences between the allergic and control mice, neither in the hippocampus nor in the parietal cortex, of either Balb/c (Fig. 6) or C57B6 (data not shown) animals.

## Discussion

In the present study, we have demonstrated that peripheral inflammation associated with a chronic model of airway-induced allergy modifies the immune status of the brain, as reflected by increased intracerebral levels of IgG and IgE, enhances tau-phoshorylation, but does not affect the APP levels, in the brain of two different mouse strains, Balb/c and C57B6. As similar findings were obtained in both mouse strains, we will thus make no further reference to the genetic background of the animals in the Discussion.

Of great interest are the increased levels of IgG and IgE in the brain of allergic mice, in comparison with the very low basal levels seen in the control mice, as determined by immunoblotting. This finding is supported by our immunohistochemical studies, in which the blood vessels were perfused with PBS prior to fixation, eliminating the blood and blood cells as a possible contaminating source of Igs. IgG is known both for its involvement in inflammatory reactions in several diseases, and also for its efficiency as an anti-inflammatory therapy in an increasing range of inflammatory disorders of the nervous system [32].

Immunohistochemical evidence indicates the presence of IgG in the brain of normal rodents [33, 34]. IgG-positive microglia has been shown to have a widespread distribution pattern in the brain of several mouse strains, including Balb/c and C57B6 [34]. IgG seems to be a normal constituent of the mammalian brain, with a concentration around 20 μg/ml in the CSF [35]. Monomeric IgG can trigger a low-grade pro-inflammatory signalling pathway at physiological levels by activating Ig Fc receptor γR1 (FcγR1) on microglia, resulting in enhanced microglial recycling endocytosis, which has been suggested to have a neuroprotective effect [35]. Microglial cells can work in a pro-inflammatory fashion, which is effective against pathogens, but a chronic inflammation, perhaps due to an endogenous origin of the process (such as ongoing neurodegeneration or amyloid pathology), may result in tissue destruction. The alternative phenotype of microglia is anti-inflammatory, and is considered protective in the case of neurodegeneration [36].

Although the functional effects of the increased levels of Igs observed in the brain of allergic animals are not known, an effect on microglia through the interaction with microglial Fc receptors may be expected. We did not find up-regulation of glial activation markers, but this does not exclude binding of Igs to microglial Fc receptors. There are several possible explanations for the lack of microglial activation observed in the present study. First, IgG may induce a low-grade inflammatory response followed by functional changes of microglia, but not by visible morphological changes. Second, it cannot be excluded that a morphologically detectable, but reversible, activation of microglia was induced at earlier time points during the allergy protocol.

Recent studies demonstrated the presence of Fc receptors on dorsal root ganglion cells, in addition to microglia [37]. Considering that neuronal Fcγ and Fcε receptors have been shown to be functional and to be activated by antigen it was suggested that IgG-, and possibly IgE- dependent activation of neurons occurs [38, 39], mediating neuroimmune communication and opening new directions in the research of neurogenic inflammation and allergic diseases. If the presence of functional Fc receptors is confirmed in the future on other neuronal cell types in the central nervous system (CNS), the effects of the increased brain levels of Igs are expected to be even more complex, hence opening for speculation on potential links to neurodegenerative disorders including AD.

The biological significance of the increased levels of Igs observed in the chronic allergy model is not obvious. However, increased IgG levels have been observed in the brain of aged individuals and Ig-positive neurons were found in the brain parenchyma of patients with AD [40]. In some studies, IgG-positive neurons were observed to have neurodegenerative apoptotic features rarely observed in the neighbouring Ig-negative neurons [40, 41]. Ig-positive neurons were found to be surrounded by a higher number of activated microglia, suggested to mediate degeneration by means of the antibody-induced classical complement pathway [41]. The intrathecal synthesis of Igs is common in neuroinflammatory diseases such as MS, in which the antigen is located within the CNS. Interestingly, intrathecal synthesis of IgG and IgM was shown to occur also in some patients with AD [42, 43]. Blennow *et al*. [42, 43] suggested that the disease process *per se* leads to development of autoantibodies through the production of tissue damage. More specifically, an immune reaction would be induced by brain tissue components (*e.g*. synapse-membrane components, glycolipids) or by disease-specific substances (*e.g*. Aβ-peptide), and released during the degenerative process [42, 43]. The induction of IgE in the brain of allergic mice in our study seems to mirror the peripheral allergic state but the consequences on the brain functionality remains to be further elucidated. Taken together, the observed increases in the brain IgG and IgE may induce a certain immunological phenotype of the brain associated with chronical airway-induced allergy. The source of IgG and IgE in the brain of the allergic mice is unknown. Igs secreted in the periphery may enter into the brain through the BBB, or peripheral Ig-secreting cells may enter the brain parenchyma. To address this question we analysed the occurrence of plasma cells in the brain by using CD138 antibodies. The immunohistochemical studies, performed on perfused animals, did not reveal any plasma cells in the brain, indicating that the Igs in the brain either originate from another cell type within the brain, or are transported over the BBB. It has been suggested that IgG enters the brain from the blood through a yet unknown mechanism, and is taken up by microglia through a Fcγ-mediated mechanism [34]. Analysis of the spleen showed a consistent increase in the number of CD138-positive cells in the allergic animals, thus confirming both the validity of our allergy model as well as the functionality of the antibody.

Although it seems reasonable to assume that our model of chronic airway-induced allergy is associated with a peripheral inflammatory response, which further affects the inflammatory status of the brain, we did not find any increase in the examined brain regions, *i.e*. hippocampus and parietal cortex, neither with regard to cellular markers specific for astrocytes and microglia nor to biochemical markers specific for Th1-Th2 responses. It has previously been shown that experimental paradigms in which an acute peripheral inflammation is elicited, for example following i.p. administration of LPS in rodents, are followed by an inflammatory response in the brain, as shown by an increase in the levels of IL-1α, IL-1β, IL-1 receptor antagonist (IL-1ra), IL-6 and TNF-α in the hippocampus and other brain regions [44–46]. Most of these studies have used high, pyrogenic levels of LPS, but even low-grade systemic inflammation induced by i.p. administration of sub-pyrogenic doses of LPS has been followed by increased expression of IL-1β, IL-6 and TNF-α in the hippocampus [46]. Moreover, it has been observed that the increased mRNA levels 2–3 hrs after LPS-challenge has a tendency to return to baseline 24 hrs after the challenge, at least for several of the cytokines [47]. A recent study involving intranasal challenge with OVA in rodents showed an induction of mRNA for the Th2 cytokines IL-4, IL-5 and IL-13 in the prefrontal cortex and olfactory bulb, although no changes in cytokine mRNAs were found in the hypothalamus and temporal cortex at 24 hrs after the last OVA-challenge [27]. We cannot exclude neither that an increase in certain cytokines may have occurred in other brain regions than the hippocampus and parietal cortex upon the chronic allergy model, nor that there is an increase in transcription to mRNA. However, the chronic nature of the allergy protocol extending over 2 months may also explain the lack of measurable variations in the levels of different cytokines, so that previous studies rather represent an acute response to peripheral challenge that may later, at least in some brain regions, be down-regulated to more normal levels. This explanation is supported by a previous study comparing the two mouse strains used in this study, Balb/c and C57B6 in acute and chronic airway-induced allergy models [30].

Another interesting finding is the increase in tau-phosphorylation observed in the allergic mice, in line with previous publications reporting that both peripherally and centrally mediated inflammatory responses increase the levels of tau-phosphorylation in the brain. Thus, peripheral inflammation associated with i.p. administration of LPS in 3xTgAD mice [48], as well as central inflammation induced by intracerebroventricular (i.c.v.) administration of LPS in a mouse model of tauopathy [49], have been shown to augment tau-phosphorylation in the brain. We have detected an increase in tau-phophorylation both at the AT8 and AT180 phospho-epitopes, known to be abnormally highly phosphorylated disease-specific epitopes in AD [50], as well as among the earliest sites to be phosphorylated [51, 52]. It can be speculated that a vicious circle may develop, in which inflammation accentuates tau-pathology, which in turn promotes additional inflammation, as it has been shown that hyperphosphorylated tau induces activation of microglia and leucocyte infiltration in transgenic rodent models of taopathy [53].

To elucidate the mechanisms that may be responsible for the observed increase in tau-phosphorylation several tau kinases were analysed. However, there were no significant changes in the levels of p-ERK, cdk5 and its regulator p35/25 or p-GSK3β, JNK or p38. It has been shown previously that the levels of several tau kinases may be altered by inflammatory factors. Intracerebral administration of LPS was shown to induce activation of cdk5 in the 3xTgAD mice [48], whereas IL-6 induced tau-hyperphosphorylation through an increase in intraneuronal levels of p35 activator, and in cdk5 activity [54]. Furthermore, LPS-induced microglial activation promotes tau-hyperphosphorylation that is dependent on functional toll-like receptor (TLR) 4 and IL-1 receptors [55]. The lack of effects on the tau kinases analysed in the present study may be due to the extended protocol of almost 2 months of treatment in the model used, during which time certain tau kinases may have been transiently increased.

Analysis of the levels of APP did not reveal any effect by the chronic allergy, neither in the Balb/c nor in the C57B6 mice. Our results are in line with recent studies in which experimentally induced inflammation in the brain has opposite effects on amyloid- and tau-pathology. Thus, peripheral inflammation associated with LPS administration in mouse models of AD has been shown to diminish amyloid-pathology, whereas tau-pathology was shown to be increased [48, 49, 56–58].

The relevance of our study is given by the fact that such a highly prevalent disease as allergy, affecting around 20% of the population, seems to be associated with modifications of the immune status of the brain. We may speculate that this could lead to irreversible effects on the brain in the form of increased tau-phosphorylation, although the connection with AD pathogenesis seems to be quite weak considering the lack of changes in APP and total tau. Regarding limitations of the study, we have yet to find the direct link between the chronic airway allergy model and the observed increase in tau-phosphorylation. Furthermore, the origin of the increased levels of Igs in the brain needs clarifying, and the biological significance of this finding is also not obvious. Despite these limitations, our study opens new directions for the research on interactions between the immune and nervous system in general, as well as for the research on the immune mechanisms involved in AD in particular.

## Supporting information

**Figure S1** The effects of chronic airway-induced allergy on the estimation of the total number of cells and of eosinophils in the bronchoalveolar lavage (BAL) fluid from Balb/c (**A**) and C57B6 (**B**) mice.

**Figure S2** The effects of chronic airway-induced allergy on the brain levels of IgG (**A** – **E**) and IgE (**A**) as shown by immunoblotting (A) and immunohistochemistry (**B**–**E**) in C57B6 mice.

**Figure S3** The effects of chronic airway-induced allergy on the levels of TH1/TH2 cytokines (interleukin (IL)-1b, -2, -4, -5, -8, -10, -12, interferon (IFN)-γ, tumour necrosis factor (TNF)-α), in the hippocampus (**A**) and parietal cortex (**B**) of C57B6 mice, as measured by Mesoscale assay.

**Figure S4** The effects of chronic airway-induced allergy on the expression of glial fibrillary acidic protein (GFAP) in the hippocampus of C57B6 mice as determined by immunoblotting.

**Figure S5** The effects of chronic airway-induced allergy on tau-phosphorylation at the AT8 (**A** and **B**) and AT180 (**C** and **D**) phosphorylation sites in the hippocampus (**A** and **C**) and parietal cortex (**B** and **D**) of C57B6 mice.
