# Supplementary figures and images for: Allergy influences the inflammatory status of the brain and enhances tau-phosphorylation
Source: J Cell Mol Med. 2012 Sep 26;16(10):2401–12. doi: 10.1111/j.1582-4934.2012.01556.x (PMC3823434; doi:10.1111/j.1582-4934.2012.01556.x)

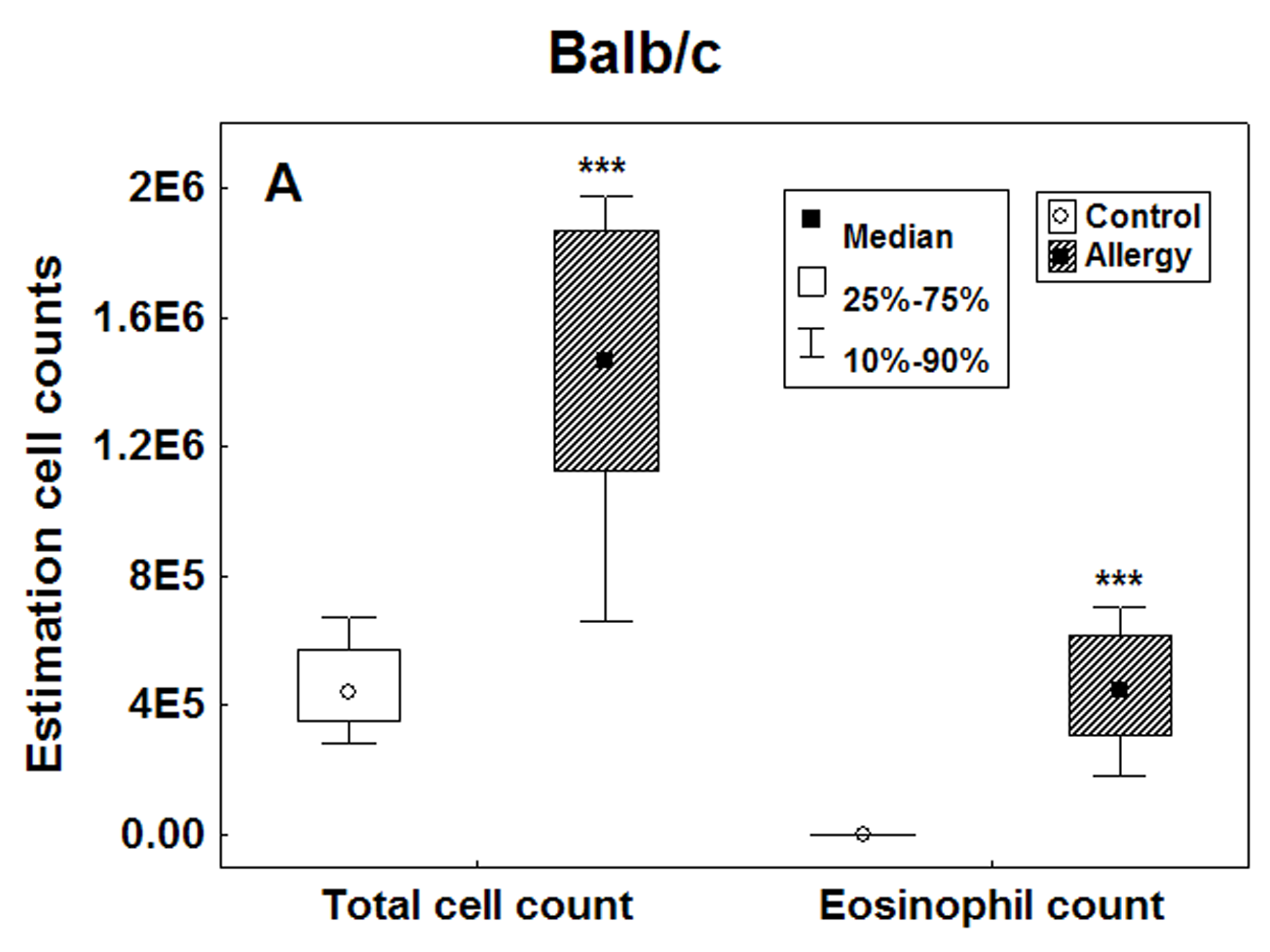

Supplement: Supplementary file 1 [file jcmm0016-2401-SD1.tif]

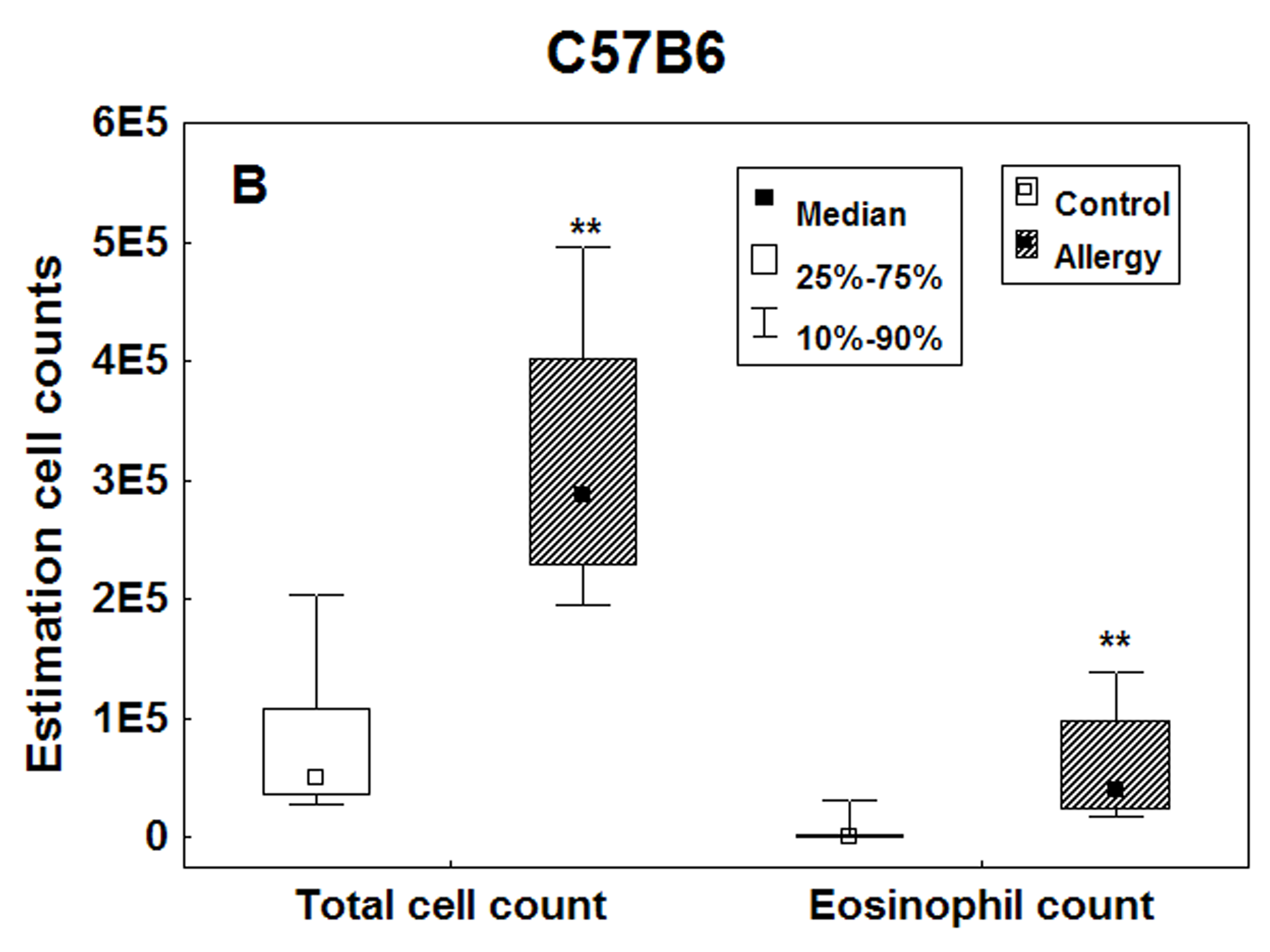

Supplement: Supplementary file 2 [file jcmm0016-2401-SD2.tif]

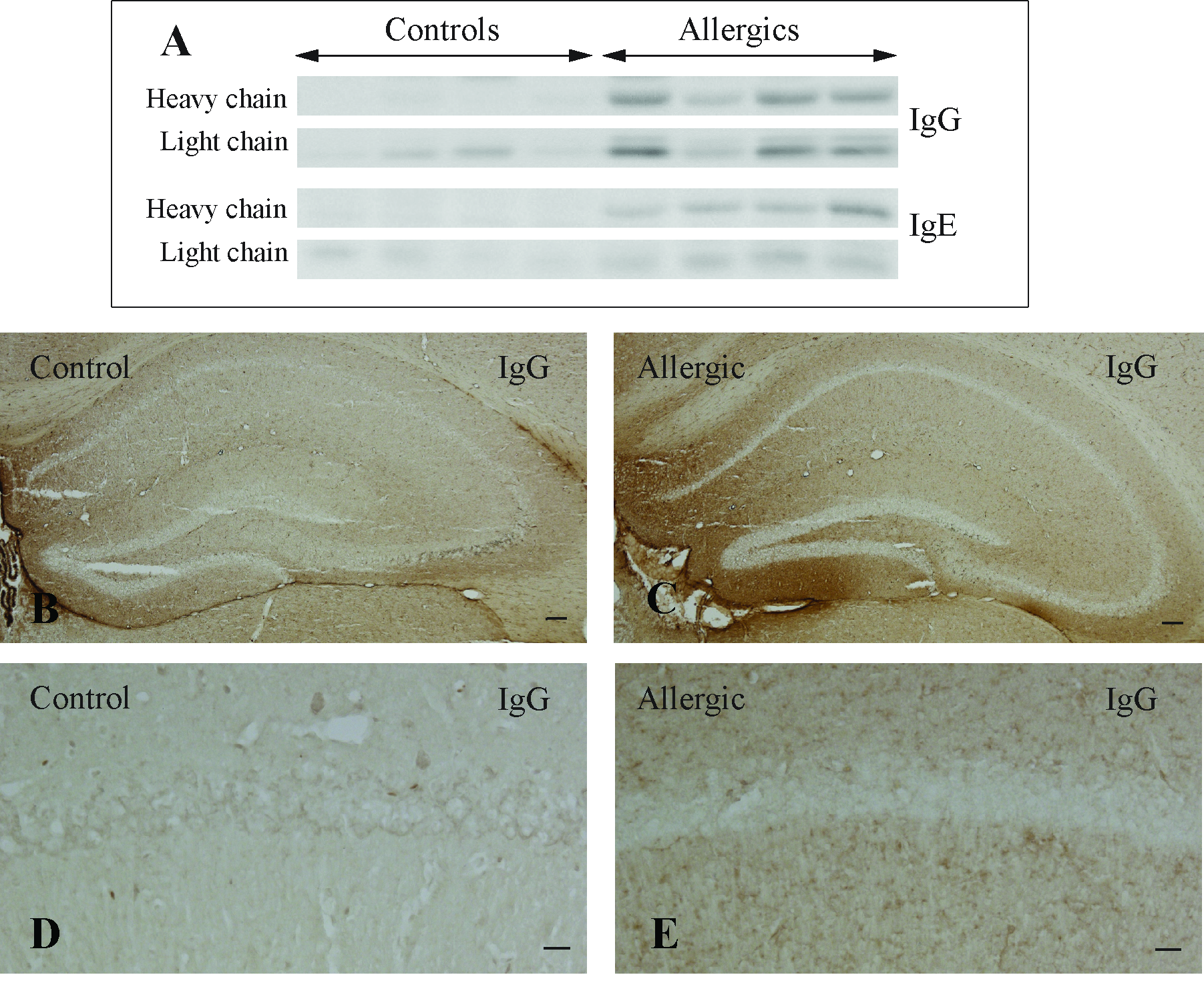

Supplement: Supplementary file 3 [file jcmm0016-2401-SD3.tif]

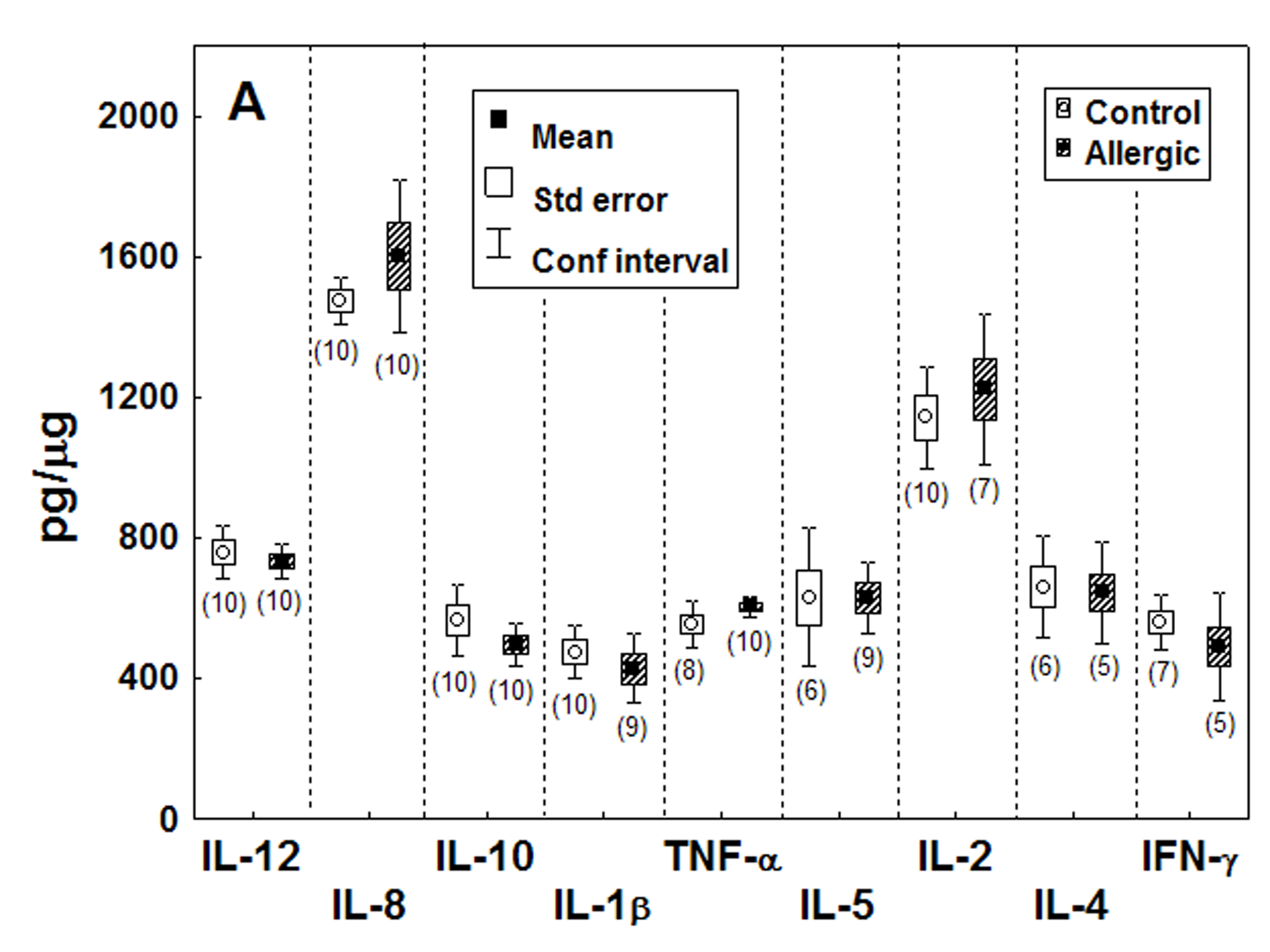

Supplement: Supplementary file 4 [file jcmm0016-2401-SD4.tif]

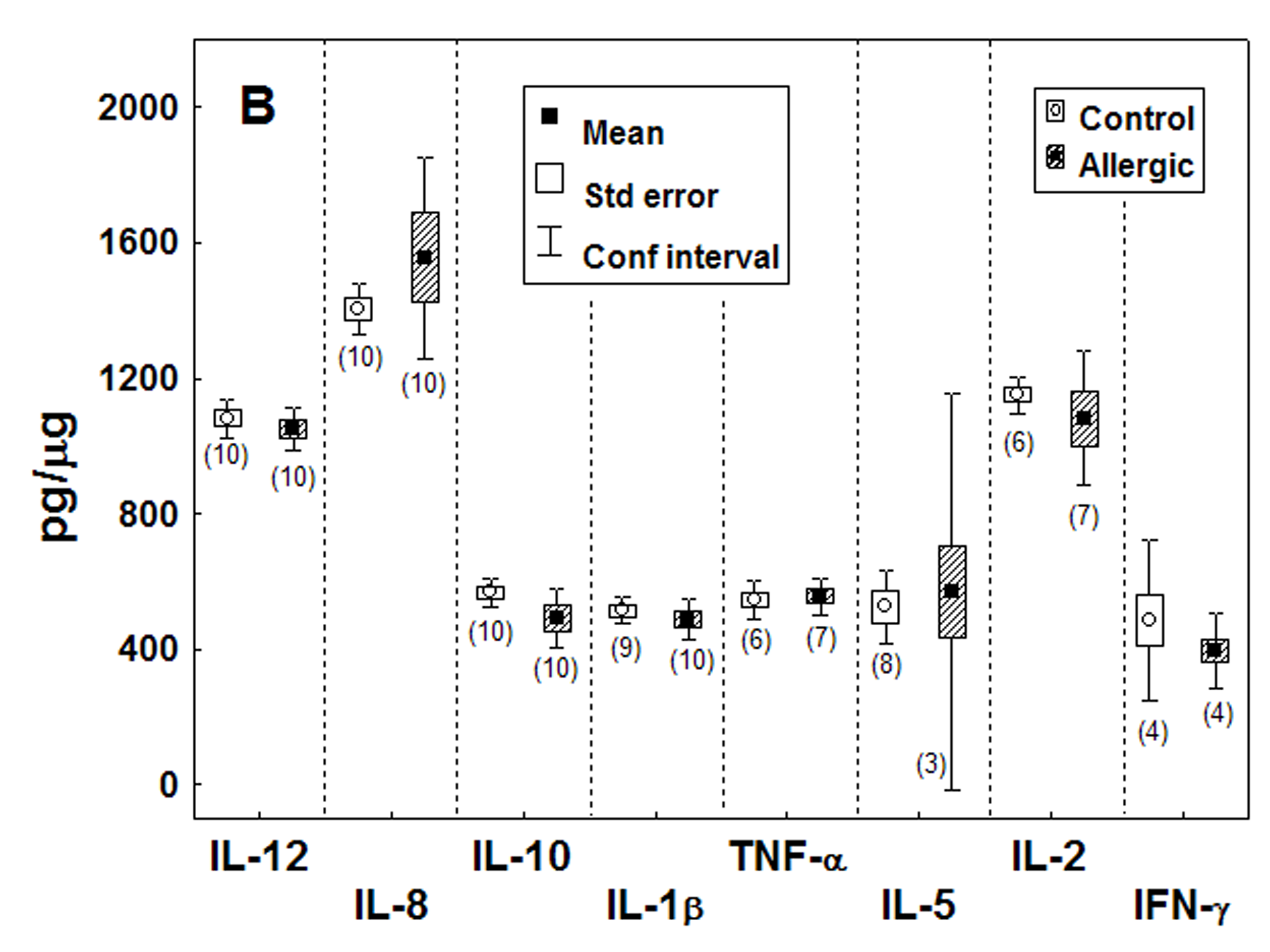

Supplement: Supplementary file 5 [file jcmm0016-2401-SD5.tif]

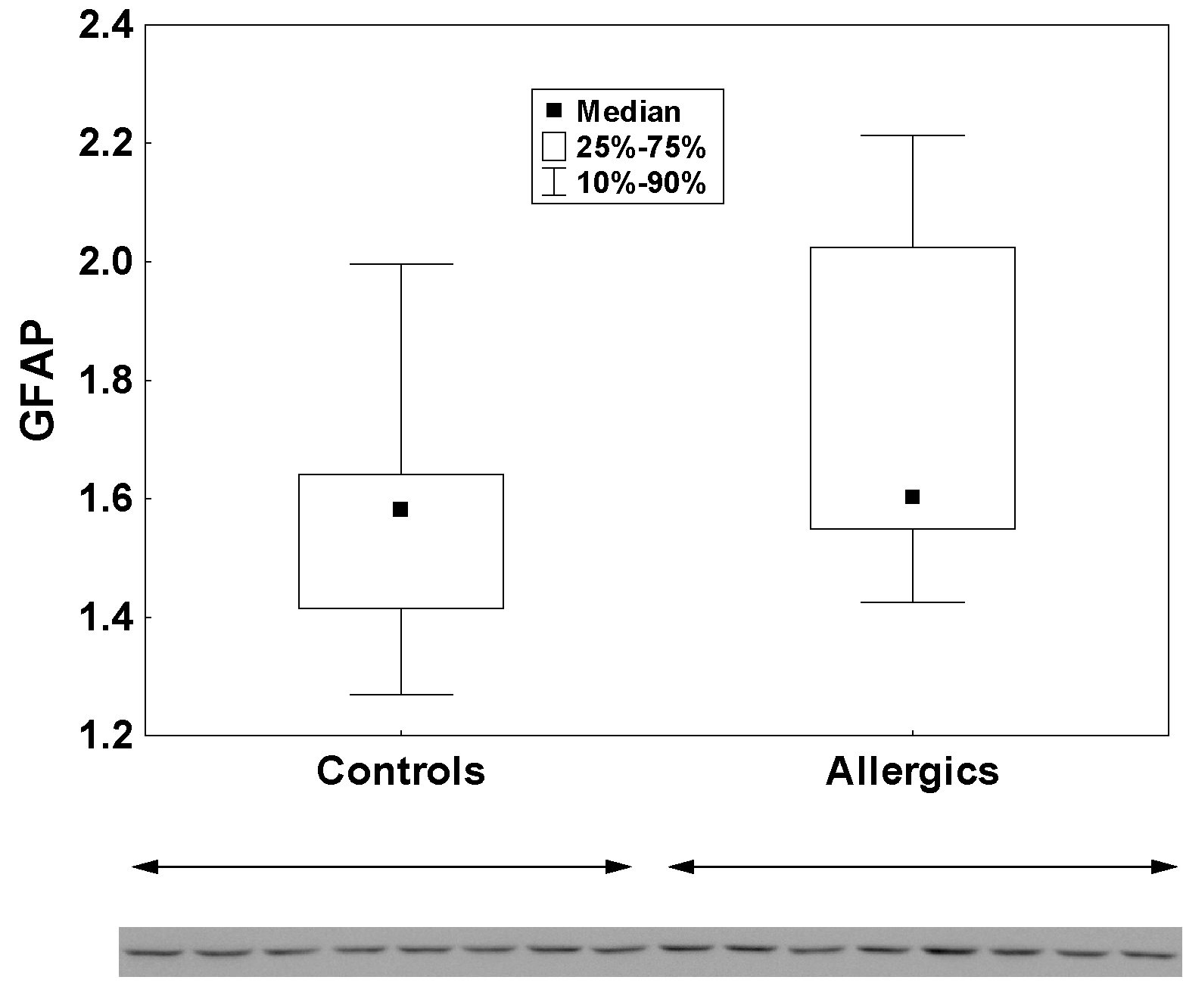

Supplement: Supplementary file 6 [file jcmm0016-2401-SD6.tif]

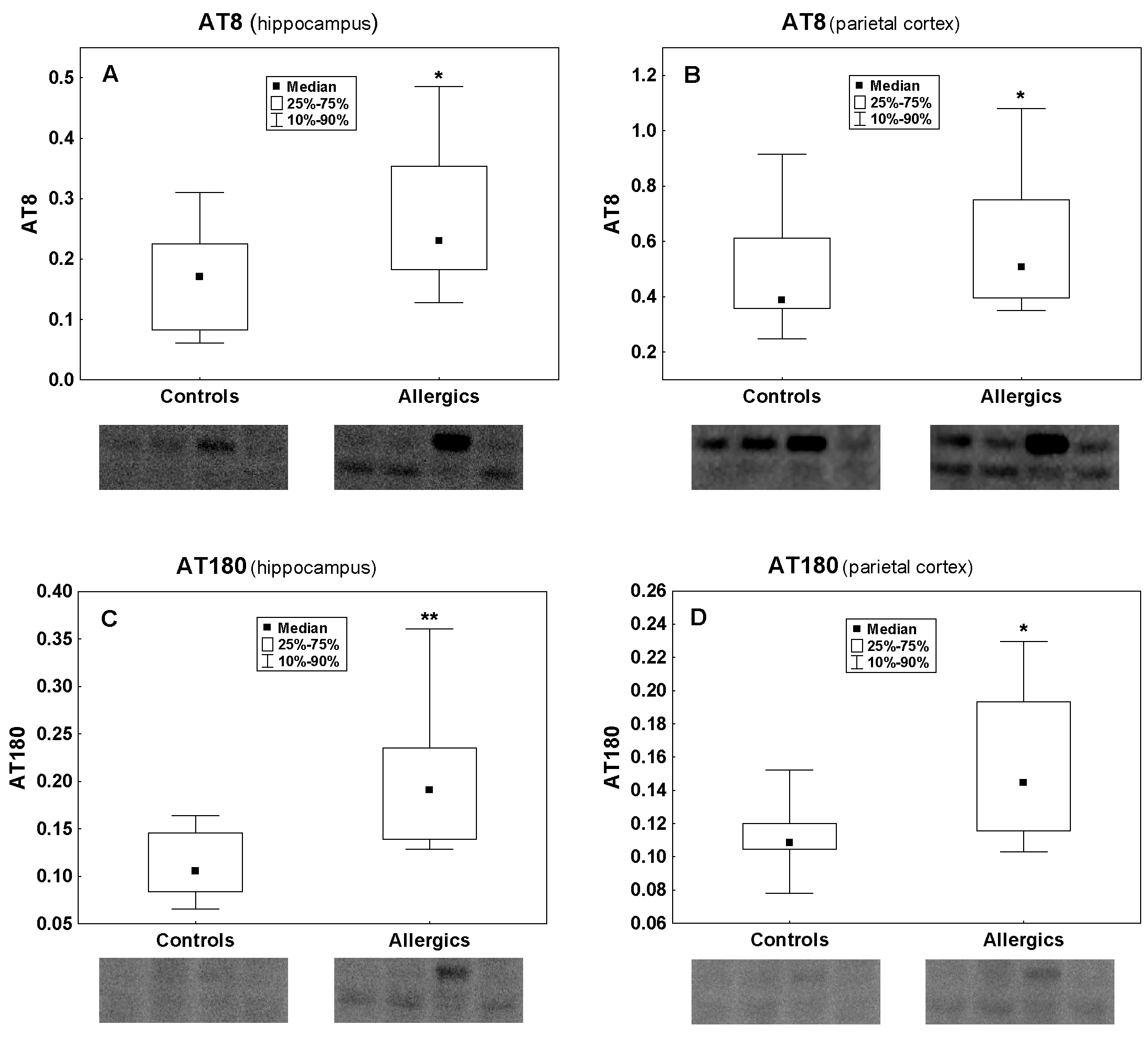

Supplement: Supplementary file 7 [file jcmm0016-2401-SD7.tif]
